# Finding Communities by Their Centers

**DOI:** 10.1038/srep24017

**Published:** 2016-04-07

**Authors:** Yan Chen, Pei Zhao, Ping Li, Kai Zhang, Jie Zhang

**Affiliations:** 1 Center for Intelligent and Networked Systems, School of Computer Science, Southwest Petroleum University, Chengdu 610500, P.R. China; 2 NEC Laboratories America, Inc. 4 Independence Way, Princeton, NJ 08540, USA; 3 Center for Computational Systems Biology, Fudan University, Shanghai 200433, P. R. China

## Abstract

Detecting communities or clusters in a real-world, networked system is of considerable interest in various fields such as sociology, biology, physics, engineering science, and interdisciplinary subjects, with significant efforts devoted in recent years. Many existing algorithms are only designed to identify the composition of communities, but not the structures. Whereas we believe that the local structures of communities can also shed important light on their detection. In this work, we develop a simple yet effective approach that simultaneously uncovers communities and their centers. The idea is based on the premise that organization of a community generally can be viewed as a high-density node surrounded by neighbors with lower densities, and community centers reside far apart from each other. We propose so-called “community centrality” to quantify likelihood of a node being the community centers in such a landscape, and then propagate multiple, significant center likelihood throughout the network via a diffusion process. Our approach is an efficient linear algorithm, and has demonstrated superior performance on a wide spectrum of synthetic and real world networks especially those with sparse connections amongst the community centers.

Many real-world systems take the form of networks in which the functional units can be considered as nodes or vertices, which are connected by links depending on their interactions. One of the most prominent features of a network is its community structure, i.e. the organization of vertices in groups, with more interactions amongst the same group than between its group members and the reminder of the network.

The community structures are closely associated with functions of specific network, thus identifying such structures yields insights into the functional organization of the network. However, finding communities within an arbitrary network can be a computationally difficult task. A growing number of community detection methods have recently been proposed since the seminal work by Girvan and Newman^1^. One popular criteria is to optimize the modularity measure^2^,^3^,^4^,^5^, like the Louvain algorithm^6^ and the Fastgreedy algorithm^7^. More recent advances involve machine learning techniques such as seeding and semi-supervised learning method^8^, neural network approaches^9^,^10^, and Bayesian^11^,^12^. For more recent developments in community detection, see^13^,^14^,^15^,^16^,^17^,^18^. Modularity measures internal connectivity of communities and uses the randomized null model as the reference. However, random networks have been found to show high-modularity subsets^19^. Moreover, for general networks, there exists a resolution limit below which modularity based methods cannot find the communities^20^.

In general, community detection falls in the scope of clustering^5^,^21^,^22^,^23^,^24^. A key concept in clustering is the measure of similarity, which to a large extent determines the clustering result. Existing similarity measures typically include the distance between two nodes^25^, common neighbors^26^, or local paths^27^,^28^. However, one limitation of these similarity measures is that they usually do not take into account the fine local topological structures of the network, such as the connection pattern among the neighbors of a node, and the connections among the important nodes. This information is crucial in determining right community structures, and clustering without consideration of these patterns may be sub-optimal.

The same limitation applies to many existing community detection algorithms, i.e., they are only designed to identify the composition of communities, but not to unravel the detailed, local structures of communities. Here we argue that the local structures of communities can also shed important light on their detection. In this paper, we leverage the concept of node density in a network, and exploit the resultant distance landscape to devise an effective algorithm that simultaneously detects communities and their centers. Our basic idea is to design a community centrality indice to quantify the relative significance of a node with respect to its neighbors in the community. Nodes with higher community centrality indice are more likely to be centers in some communities. Based on the election of the central nodes in the communities, we are then able to categorize the reminder of nodes into communities using an iterative and greedy propagation strategy. This strategy resembles a multi-source diffusion and decision-making process which is simple with low complexity.

We show that by incorporating the local topological, structural modeling in the process of community detection, our approach can detect communities more accurately in several benchmark systems including both synthetic and real-world scenarios against state-of-the-art. The superiority of our approach is particularly significant for networks with their centers far away from each other. However, for some networks in which the centers may have more connections among them, it can be challenging for our approach to identify exact communities due to the less clarified boundaries between communities.

Very relevant to our work is that of Rodriguez and Laio^29^, who presented an efficient clustering approach for data points in the Euclidean space. The basic idea is that these cluster centers are those surrounded by more points (so-called density) than their neighbor points and they have relatively large distance from each other. This algorithm need not be an iterative procedure and thereby is very fast. In comparison, however, the task of community detection is to cluster nodes in the topological space, which is very different from data clustering in many respects. Topological properties and local connection profiles must be considered for reliable community detection in complex networks or graphs.

## Results

We test the performance of our method on both synthetic and real-world networks by comparing the outcome of our algorithm with the ground-truth community structures and results of other community detection methods. The synthetic networks are generated by the LFR-benchmark model^30^, which produces networks with power-law degree distribution and with implanted communities within the networks, and the real-world networks are of different types and different scales. The networks used in our experiments are shown in Table 1.

### Synthetic Networks

To demonstrate the effectiveness of the proposed method, we generate four benchmark networks using procedures presented in ref. 30 and the detailed parameters are shown in Table 1. Among the 4 artificial networks, three of them have clear, non-overlapping community structures; and the other network has 5 overlapping communities. We use the Normalized Mutual Information (NMI)^31^ to measure the performance of different algorithms on detecting communities of these networks.

**Figure 1:**
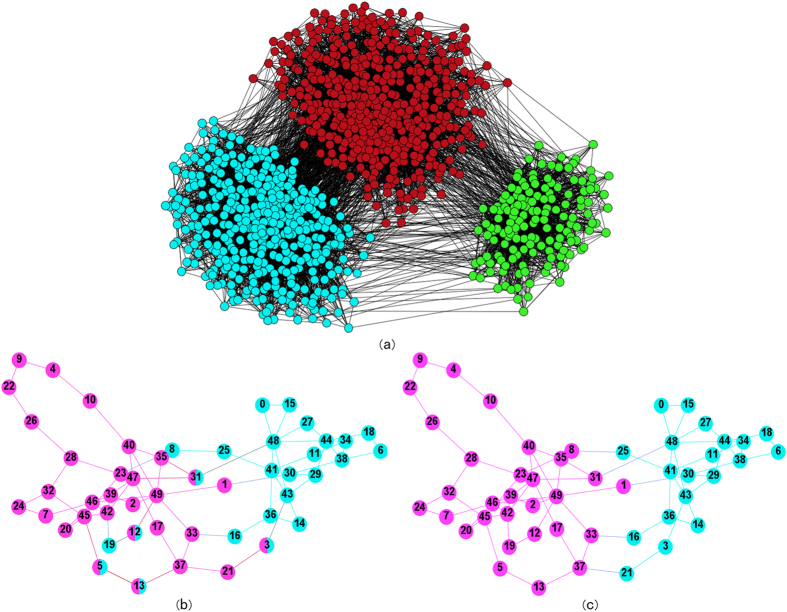
Two networks of LFR-benchmark. (**a**) LFR-2 with 1000 nodes and three communities. The result of our algorithm agrees with the ground truth. (**b**) The real communities of LFR-4 given by the LFR benchmark algorithm. 5 overlapping nodes are shown using pie vertex in two different colors. (**c**) Our partition of LFR-4.

As can be seen from Table 2, for the first three networks with completely disjoint communities, our approach achieves a 100% accuracy in identifying actual communities. The communities in the fourth network generated from the model is shown in Fig. 1(b) and our result is shown in Fig. 1(c). It is obvious that the overlapping nodes in the benchmark are not quite reasonable, while our approach can properly partition the nodes into explicit two parts.

### Real-world Networks

**Figure 2:**
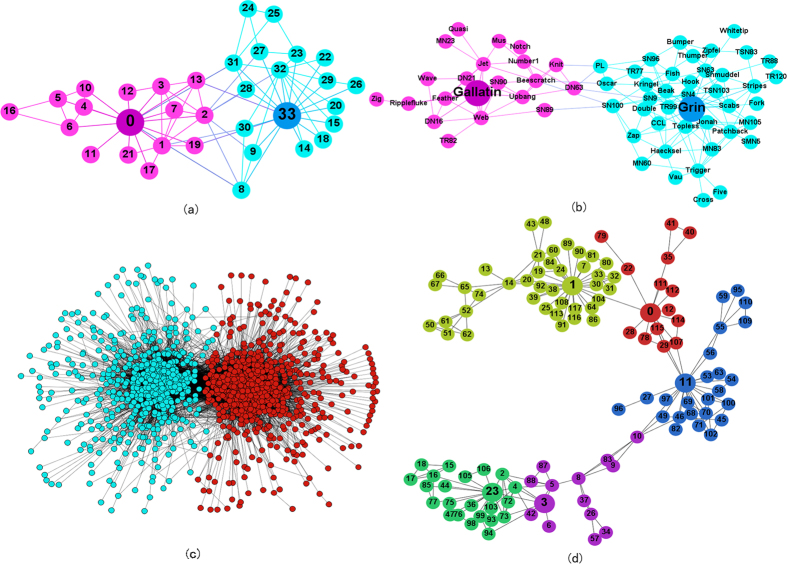
The partition results by the proposed method for real-world networks. (**a**) Zachary karate club network: the two communities we detected are identical with the real communities. (**b**) Dolphins network: the 2 communities we detected are identical with the 2 real groups of male and female. (**c**) Pol-blogs network: the 2 communities we discovered. (**d**) The SFI collaboration network: this network has obvious tree structure, the degree density indice can be used to find the centers.

Several real-world networks are used to test the validity of our algorithm. The first one is Zachary karate club network^32^ which is a famous de facto network. A conflict between club president John (node 33) and the instructor Mr. Hi (node 0) leads to 34 members of the university sports club to split into two groups. Figure 2(a) shows that the communities discovered by our algorithm agree exactly with the result given by Zachary^32^. The leaders of the two groups are node 0 and node 33, which is consistent with the ground truth too.

The second network is the social network of bottlenose dolphins reported by Lusseau^33^, which is an undirected social network of frequent associations between 62 dolphins in a community living off Doubtful Sound. In this network, dolphins are represented as vertices, and a link is attached between two nodes if the corresponding dolphins are observed together more often than expected by chance over a period of seven years from 1994 to 2001. The groups of dolphins are mainly divided into the male ones and female ones. Our result is shown to be completely the same as the ground truth. The two communities are marked by purple and blue, respectively (shown in Fig. 2(b)).

The third network is the political blogs data-set which is a directed network of hyperlinks between weblogs on US politics, recorded in 2005 by Adamic and Glance^34^. The network is separated according to the political orientation of blogs, conservative or liberal. Due to the unconnectedness of the original network, we consider the undirected version of the network and retrieve the maximum component to detect communities. The maximum component has 1222 nodes and 16717 edges. The diameter is 8 and the average shortest path is 2.858. The NMI between our identified communities and the ground truth is 0.72. The visualization of communities detected is shown in Fig. 2(c).

The fourth network is the SFI collaboration network with 271 scientists at the Santa Fe Institute^1^, an interdisciplinary research center in Santa Fe, New Mexico, with the largest component consisting of 118 scientists. An edge is drawn between a pair of scientists if they coauthored one or more articles. The network includes all journal and book publications by the scientists involved, along with all papers that appeared in the institute’s technical reports series. The network has several hub nodes with high degrees. In this network, we have tried different types of density indices in the implementation of our algorithm. We first consider the “strong-tie” density as the measure to select the central nodes, leading to a modularity of 0.65. We then use the classical degree density to select nodes as the centers of communities, and the resultant modularity is 0.70, showing the effects of different local centralities on the performance. The partition result by the degree-density is shown in Fig. 2(d).

We also test our method on several networks without ground-truth community partitions, with the size of the networks ranging from a hundred to tens of thousands, see Table 3. The comparison with other approaches is provided in the next section.

### Comparison With Other Methods

To further assess our method, we compare our partition results with five popular algorithms: Louvain, Fastgreedy, Infomap^35^,^36^, Eigenvector^37^ and Label propagation (LP)^38^, using NMI and modularity^39^ as the evaluation metrics. From Table 2, we can see that for networks with ground truth communities, our method is better than or similar to other algorithms using the NMI criterion, except on the American football network.

For those networks without the ground truth information (the networks in Table 3), we use the modularity to measure the quality of community detection results. It can be seen that the modularity values of our partitions are lower than those obtained by the Louvain and Fastgreedy algorithms. This can be expected because our method is not specifically designed to optimize the modularity as Louvain and Fastgreedy algorithms do. However, the modularity values obtained by our method are almost always better than the other three algorithms, i.e., Infomap, Eigenvector and Label propagation, especially for large sparse networks with low rich-club connectivity (e.g. PGP, CA-HepTh and Power Grid), see Table 3.

## Discussion

We present a simple and novel method to detect community structures in complex networks. In our approach, a structural central node in each community will be determined firstly, and other nodes will be partitioned into different communities according to a multi-source diffusion and majority voting process (see detailed discussions in Methods). Compared with popular algorithms in the literature, our algorithm has robust performance in both synthetic and real-world networks with which ground truth is known, and the modularity results is also competitive in most of the networks. The whole algorithm has a linear time complexity, which fits it for large-scale problems.

From Table 3, we can also observe that for some networks, such as Jazz and Wiki-vote, the modularity obtained by our approach could be inferior to several other methods under comparison. We speculate that in these networks, there exist dense connections among detected community centers, therefore it becomes more challenging to identify exact communities. On the contrary, for networks whose community centers demonstrate sparse inter-connections among each other, our approach is expected to produce significant performance gains (e.g. PGP, CA-HepTh and Power Grid).

In general application scenarios, we can use the rich-club connectivity of the network^40^,^41^ as an indicator of the performance of our approach. The rich-club connectivity is an interesting property that describes the amount of linkages among “rich” nodes of a network (i.e., nodes with high degrees, which are very likely the community centers). Typically, the lower the rich-club connectivity, the sparser the inter-connections among the center nodes, and hence our approach is expected to give a better performance; on the contrary, the higher the rich-club connectivity, the denser the inter-connections among the center nodes, and our approach may give inferior results.

To validate this indicator, we have included the rich-club connectivity of different networks in Table 1. Note that the rich-club connectivity *ϕ*(*r*) is a function of *r*, where *r* is the position of the node in the ordered list (from larger degrees to small degrees), normalized by the number of nodes *N*. In practice, we first choose the number of high-degree nodes *k*, and then examine whether the rich-club connectivity computed using *r* = *k*/*N* is above/below a pre-defined threshold (such as 0.5). In our experiments, *k* is chosen as a small number based on the logarithm of the number of nodes (as shown in Table 1). For example, the rich-club connectivity *ϕ*(*r*) for Jazz and Wiki-vote is 0.94 and 0.55, respectively; both of which are quite high, and as a result our approach does not produce a good modularity on these networks. In comparison, the rich-club connectivity *ϕ*(*r*) for PGP, CA-HepTh and Power Grid is lower than 0.2, and the performance of our approach on these networks are much more superior. This demonstrates the usefulness and applicability of the rich-club connectivity in predicting the performance of the proposed method.

## Methods

The key intuition in our algorithm is that the central node in a community should be highly surrounded by other members in this community, namely it has a high density; while neighbors of the central node may not connect tightly with each other. We elaborate the three steps of our method in the following subsections.

### Calculating Density Indice

We first calculate a density indice, denoted by *η*, for each node. The density indice can be defined in two ways: degree indice (*η*_*d*_) and strong-tie indice (*η*_*s*_). As we know, a node’s degree is the number of neighbors of the node. Larger degree means that the node has more neighbors and therefore it has a high local density. Strong-tie indice^42^ is defined in our algorithm as the number of triangles involving node *i*. A large strong-tie indice means that node *i* has more neighbors and its neighbors have more connections amongst themselves. The existence of such nodes strongly indicates the existence of communities. From Table 4 we can find that the performance by adopting strong-tie is better than the case of using degree in some networks but vice versa in other real networks. Our findings indicate that for large-scale networks, degree indice is more suitable than strong-tie. In the following we call both these two indices density *η*-score to simplify notations.

### Identifying Central Nodes

In this step the key objective is to remove non-central nodes based on *η*-scores. Intuitively, the central node should have a large distance to other central nodes (or nodes with higher *η*-scores); on the contrary, the central node will be relatively close to its neighbors (or nodes with lower *η*-scores). Based on this observation, we define the notion of Eta-reach-distance (ERD) *ψ* to facilitate the choice of central nodes. In particular, the ERD for *i*th node, *ψ*_*i*_, is the minimum of the shortest path distances between node *i* and all other nodes with a higher *η*-score,


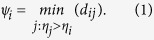


The usefulness of the Eta-reach-distance can be understood as follows. If a node *i* has a low ERD, that means *i*’s close neighbors have higher *η*-score than node *i*, then the node *i* will very unlikely be a central node; on the other hand, if the node *i* has a large ERD, then that means in order to find a node whose ERD is higher than node *i*, one has to go far away in the network, which means that node *i* is probably a central node. In fact, large *ψ*_*i*_ always appears at local or global maxima of the density scores. Given this property, we then identify the central nodes as those with particularly large *ψ*_*i*_’s.

To reduce the computational cost, in searching the neighbors of node *i*, we will apply the breadth first search strategy. We first examine the first-order neighbors of a given node *i*, and if any of them has an *η*-score larger than node *i*, we will then set *ψ*_*i*_ = 1. Otherwise, we search the second order neighborhoods and if any of them has a larger *η*-score, we set *ψ*_*i*_ = 2; if not, we will examine all the 3rd-order neighbors of node *i* until the end. In general, it is enough to search in the neighborhood at depth 2, owing to dense connectivity structures of the community. So we will typically stop at *ψ*_*i*_ = 3 if we can not find a node with larger *η*-score and search no further. It should be noted that for some networks such as Jazz^43^ and Email^44^, etc, centers of the communities are directly connected with each other based on the ground truth available. In these cases, *ψ* for most of the nodes would be 1 and cannot be used to find the centers.

**Figure 3:**
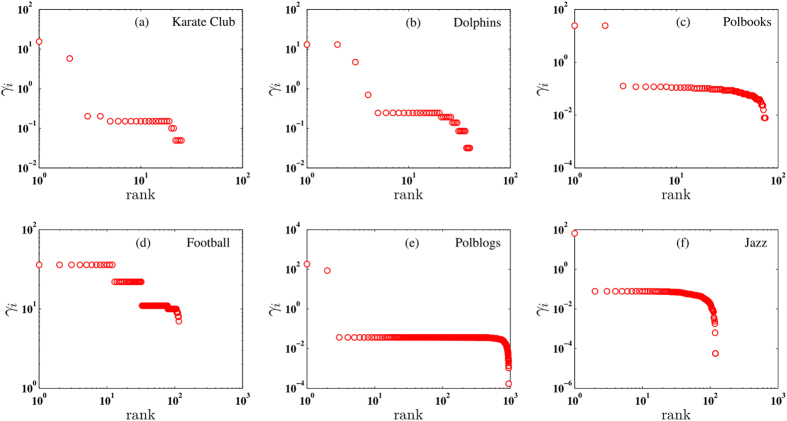
The log-log graph of *γ*_*i*_ = *η*_*i*_*ψ*_*i*_ in descending order for six small networks. *η*_*i*_ and *ψ*i are normalized so that most of the nodes are very small.

Sometimes, in order to consider the combined effect of local density and relative distance, we define the community centrality *γ*_*i*_ = *η*_*i*_*ψ*_*i*_ to depict the importance of the node in a community, as shown in Fig. 3. Then we sort the *γ* values in an descending order and choose the largest *C* nodes as the centers corresponding to the *C* communities. Consider that we do not know the exact number of communities in a network, we try several *C* values to find the best one with the largest modularity. Sometimes, we may observe an obvious turning point on the sorted *γ* values, as is shown in Fig. 3. In such circumstance we can simply use this turning point to decide which nodes should be the central nodes. For example, for the karate club data, there are two nodes with significantly larger *γ*’s than others, thus it’s better to divide the karate club network into two groups.

### Label Propagation

After the central nodes have been identified and assigned proper community labels, their labels will diffuse in the whole network such that all the rest nodes can be labeled as well. We achieve this by using majority voting, namely, any node without a community label will accept one that presents most frequently in its (labeled) neighbors. To reduce the uncertainty in label propagation, we adopt a greedy, iterative scheme. In each iteration, among all the unlabeled nodes with sufficient labeled neighbors, we will only target on that node with the largest *γ* value. By doing this, the propagation process will affect only the most confident node one at a time, which is not only computationally efficient but also improves the labeling quality.

### Complexity

Our algorithm consists of three steps. In the first step of calculating the density indice, the time complexity is O(*n*), where *n* is the number of nodes. In the second step, computing the ERD requires O(*m*) time, where m is the number of edges; sorting the *γ* values takes O(*n*) time if bucket sort algorithm is considered. The third step of community label assignment will require O(*n*) time. Thus, the total time complexity of our method is O(*m* + *n*). As a result, this algorithm has linear time complexity and can be efficiently applied to a network of tens of thousands of nodes.

## Additional Information

**How to cite this article**: Chen, Y. *et al*. Finding Communities by Their Centers. *Sci. Rep*. **6**, 24017; doi: 10.1038/srep24017 (2016).

## Figures and Tables

**Table 1:** Networks used in the experiments.

Synthetic Networks										
Networks	*n*	*k*_*m*_	*ϕ*(*r*)	*μ*	*t*_1_	*t*_2_	*c*_*min*_	*c*_*max*_	*n*_*o*_	*m*_*o*_
LFR-1	50	3	0	0.1	2	1	25	25	0	0
LFR-2	1000	15	0.18	0.1	2	0	500	500	0	0
LFR-3	1000	20	0.07	0.1	2	1	100	500	0	0
LFR-4	50	3	0	0.1	2	1	25	25	5	2
**Real-world Networks**										
Networks	*n*	*k*_*m*_	*ϕ*(*r*)	Description						
Karate	34	4.59	0	Zachary’s social network of a karate club^32^						
Dolphins	62	5.13	0.67	Dolphin social network^33^						
Polbooks	105	8.40	0.4	Books about US politics^2^						
Football	115	10.66	0.3	Network of American football games^2^						
SFI	118	3.40	0.3	Collaboration network of scientists at the Santa Fe Institute^1^						
Jazz	198	27.70	0.94	Network of Jazz musicians^43^						
E-coli	328	3.03	0.13	Transcriptional regulation network of Escherichia coli^45^						
Email	1133	9.62	0.27	Network of e-mail interchanges^44^						
Polblogs	1222	27.36	0.5	Blogs about politics^34^						
Power Grid	4941	2.67	0.02	The Western States Power Grid of the United States^46^						
Wiki-vote	7066	28.51	0.55	Wikipedia who-votes-on-whom network^47^						
CA-HepTh	9877	5.74	0.1	Collaboration network of Arxiv High Energy Physics Theory^48^						
PGP	10680	4.55	0.17	Web of trust of PGP^49^						
CA-CondMat	23133	8.55	0.28	Collaboration network of Arxiv Condensed Matter^48^						
Email-Enron	36692	10.73	0.45	Email communication network from Enron^50^						

Here *n* denotes the numbers of vertices, for networks that are not fully connected, the largest graph components are considered. *k*_*m*_ is the averaged node degree, *μ* is the mixing parameter, *t*_1_ is the negative exponent for the degree distribution, *t*_2_ is the negative exponent for the community size distribution, *c*_*min*_ and *c*_*max*_ is the minimum and maximum size of communities, respectively, *n*_*o*_ is the number of overlapping nodes, and *m*_*o*_ is the number of memberships in the overlapping nodes. The *ϕ*(*r*) is the rich-club connectivity of the network^40^. Here we choose *r* ∼ log *N*/*N*.

**Table 2:** Performance comparison in the networks with ground truth.

Networks	Ground Truth		Ours			Louvain		Fastgreedy		Infomap		Eigenvector		LP	
	C	Q	C	NMI	Q	NMI	Q	NMI	Q	NMI	Q	NMI	Q	NMI	Q
LFR-1	2	0.43	2	**1.00**	0.43	0.40	0.56	0.51	0.56	0.46	0.56	0.51	0.52	0.40	0.51
LFR-2	3	0.52	3	**1.00**	0.52	1.00	0.40	0.88	0.39	1.00	0.40	1.00	0.40	1.00	0.40
LFR-3	2	0.40	2	**1.00**	0.40	1.00	0.52	0.99	0.51	1.00	0.52	0.88	0.49	1.00	0.52
LFR-4	2	0.39	2	**0.51**	0.41	0.45	0.54	0.34	0.53	0.39	0.55	0.43	0.51	0.33	0.46
Karate	2	0.37	2	**1.00**	0.37	0.59	0.42	0.69	0.38	0.70	0.40	0.68	0.39	0.70	0.40
Polbooks	2	0.41	2	**0.60**	0.46	0.51	0.52	0.53	0.50	0.49	0.52	0.52	0.47	0.57	0.50
Football	12	0.55	12	**0.86**	0.59	0.88	0.60	0.70	0.55	**0.92**	0.60	0.70	0.49	**0.92**	0.60
Dolphins	2	0.38	2	**1.00**	0.38	0.48	0.52	0.61	0.50	0.50	0.52	0.54	0.49	0.69	0.50
Polblogs	2	0.41	2	**0.72**	0.42	0.63	0.43	0.65	0.43	0.48	0.42	0.69	0.42	0.69	0.43

Here *C* is the number of communities, *Q* is the modularity result and *NMI* the normalized mutual information.

**Table 3:** Performance comparison in the networks without ground truth.

Networks	Ours		Louvain		Fastgreedy		Infomap		Eigenvector		LP	
	*C*	*Q*	*C*	*Q*	*C*	*Q*	*C*	*Q*	*C*	*Q*	*C*	*Q*
CA-CondMat	105	0.63	55	0.72	261	0.63	1347	0.63	34	0.54	1537	0.62
Email-Enron	267	0.42	246	0.60	560	0.51	1554	0.52	2	0.34	887	0.32
CA-HepTh	60	0.76	53	0.82	79	0.78	520	0.73	22	0.57	541	0.74
PGP	121	0.82	102	0.88	190	0.85	1070	0.80	25	0.68	955	0.81
Power Grid	35	0.90	40	0.93	39	0.93	483	0.82	35	0.83	479	0.81
Wiki-vote	9	0.34	9	0.43	31	0.34	254	0.38	10	0.42	3	9e-05
Jazz	2	0.29	4	0.44	4	0.44	7	0.28	3	0.39	2	0.28
Email	19	0.43	12	0.54	16	0.51	68	0.52	7	0.49	8	0.28
E-coli	10	0.66	14	0.75	15	0.75	39	0.71	11	0.64	42	0.68
SFI coli	5	0.70	8	0.75	8	0.73	14	0.72	7	0.71	11	0.70

Here *C* is the number of communities, *Q* is the modularity result.

**Table 4:** Impact of different density indice (strong-tie and degree) on the Performance of our approach.

Networks	Strong-tie		Degree	
	*C*	*Q*	*C*	*Q*
Karate	2	**0.37**	2	**0.37**
Dolphins	3	**0.48**	3	0.43
Polbooks	7	**0.48**	3	0.44
Polblogs	2	**0.42**	2	**0.42**
Football	12	**0.59**	15	0.51
E-coli	10	**0.66**	15	0.63
SFI	3	0.65	5	**0.70**
Jazz	44	**0.33**	41	0.32
Email	18	0.41	19	**0.43**
CA-CondMat	105	0.61	145	**0.63**
Email-Enron	224	0.41	267	**0.42**
CA-HepTh	86	0.74	60	**0.76**
PGP	126	0.81	121	**0.82**
Power Grid	63	0.88	35	**0.90**
Wiki-vote	7	0.32	9	**0.34**

The results shown here are the modularity (*Q*) obtained by the proposed method and the number (*C*) of communities identified.
